# Analyzing the Complicated Connection Between Intestinal Microbiota and Cardiovascular Diseases

**DOI:** 10.7759/cureus.28165

**Published:** 2022-08-19

**Authors:** Tanishq Kumar, Rajoshee R Dutta, Vivek R Velagala, Benumadhab Ghosh, Abhay Mudey

**Affiliations:** 1 Medicine, Jawaharlal Nehru Medical College, Datta Meghe Institute of Medical Sciences, Wardha, IND; 2 Otolaryngology, Jawaharlal Nehru Medical College, Datta Meghe Institute of Medical Sciences, Wardha, IND; 3 Community Medicine, Jawaharlal Nehru Medical College, Datta Meghe Institute of Medical Sciences, Wardha, IND

**Keywords:** vascular changes, rheumatic heart disease, atrial fibrillation, stroke, cyanotic heart disease, heart failure, hypertension, atherosclerosis, gut microbiota

## Abstract

Relentless human curiosity to understand the basis of every aspect of medical science has led humanity to unlock the deepest secrets about the physiology of human existence and, in the process, has reached milestones that a century ago could only be imagined. Recent ground-breaking breakthroughs have helped scientists and physicians all over the world to update the scientific basis of diseases and hence further improve treatment outcomes. According to recent studies, scientists have found a link between intestinal flora and the pathogenesis of diseases, including cardiovascular diseases. Any change in the typical habitat of gut microbiota has been shown to result in the culmination of various metabolic and cardiac diseases. Therefore, gut microbiota can be credited for influencing the course of the development of a disease. Any change in the composition and function of bacterial species living in the gut can result in both beneficial and harmful effects on the body. Gut microbiota achieves this role by numerous mechanisms. Generations of various metabolites like TMAO (trimethylamine N-oxide), increased receptibility of various bacterial antigens, and disruption of the enzyme action in various metabolic pathways like the bile acids pathway may result in the development of metabolic as well as cardiovascular diseases. Even if they may not be the only etiological factor in the pathogenesis of a disease, they may very well serve as a contributing factor in worsening the outcome of the condition. Studies have shown that they actively play a role in the progression of cardiovascular diseases like atherosclerotic plaque formation and rising blood pressure. The focus of this review article is to establish a relation between various cardiovascular diseases and gut microbiota. This could prove beneficial for clinicians, health care providers, and scientists to develop novel therapeutic algorithms while treating cardiac patients.

## Introduction and background

According to the American Heart Institute (AHA), cardiovascular diseases (CVDs) are the highest contributor to mortality and morbidity in the world. The cardiovascular system has become the single most affected system, leading to the highest number of deaths in the USA and worldwide [1]. Scientists are constantly maximizing their efforts to find novel therapies and interventions to reduce this global burden of CVDs. Not very long ago, various studies found an association between intestinal flora and its role in the pathogenesis of many diseases like intestinal pathologies, cardiovascular conditions (CVDs), and metabolic disorders [2]. The inception of gut colonization by microbiota starts at the level of an infant as the fetal gut is believed to be sterile and only a few species of microbes are found in an infant. As the infant grows and interacts with external factors, this leads to increased gut microbiota habitation. Therefore, many species inhabit and thrive in the gut, resulting in the homeostatic intestinal flora. This happens provided there are no pathological interventions for the growing gut flora populations. Any environmental or behavioral factor leading to an alteration or a change in the habitat of the intestinal flora is known as Dysbiosis. This change results in minor pathologies like inflammation, disruption in enzyme function, etc., ultimately leading to various pathological conditions [3,4]. 

Intestinal flora or Gut microbiota is a generalized term for all the species inhabiting the gut/intestines. It includes both the species which are beneficial and detrimental to our health. A correct balance of both types is needed for the proper maintenance of the ecological system in the gut. They are very significant in boosting intestinal immunity as well as regulating various physiological and chemical reactions [5]. Few species of anaerobic bacteria contribute significantly to the intestinal flora, which include *Bacteroidetes*, *Firmicutes*, *Proteobacteria*, *Actinobacteria*, *Fusobacteria,* and *Verrucomicrobia* [6-9]. These species and several other families of microbes are extensively involved in carrying out various physiological activities. The proper external environment, consumption of the right diet, justified drug use, mental health, and other factors are vital for maintaining the correct composition of the bacterial species. Any intervention leading to intestinal flora disturbance might lead to disruption of various pathways, eventually contributing to the development of various diseases. Therefore, it is the need of the hour to conduct studies that would further explore the association between microbiota and the pathogenesis of various diseases. This review tries to broaden our knowledge about gut microbiota’s role in numerous cardiovascular diseases.

## Review

Understanding gut microbiota’s role in the human body

The gut is inhabited by billions of microbes that have both beneficial and harmful effects. The primary function of the gut microbiota is to aid the digestion of macromolecules that we consume in our diet. This is achieved by proteolytic and saccharolytic mechanisms [10]. The proteolytic pathway involves the breakdown of protein and peptides, generating short-chain fatty acid (SCFA) as a by-product. Similarly, the breakdown of carbohydrates is done by the saccharolytic pathway, which also contributes to the release of short-chain fatty acids. SCFA contributes to the development of CVDs. Some other by-products like microbial uremic toxins are also released, which can hamper renal physiology [11].

Colonies of different microbes are responsible for various actions occurring in the body and are not just limited to intestinal digestion. Maintaining the integrity of the intestinal lumen, acting against the proliferation of harmful species, role in the absorption of nutrients, and immunological response of the gut are some functions that come under the purview of gut microbes. Therefore, gut microbiota forms the core of gut functions and plays a significant role in the overall well-being of an individual [12-17].

Coronary artery disease

According to a recent report, coronary artery disease (CAD) contributed to about 12.6% of deaths in the United States in 2018. This data indicates that every 40 seconds, one person dies due to CAD [1]. Coronary artery disease is developed when there is a partial or complete blockage in the coronary artery leading to myocardial ischemia if unresolved leads to myocardial infarction [18]. According to studies, similar families of microbes were present in both the plaque as well as in the intestinal flora of the same individual. This possibly shows some relation between gut microbiota and the pathogenesis of CAD. *Chryseomonas*, *Collinsella*, *Veillonella,* and *Streptococcus* species are found to be in a higher concentration in atherosclerotic patients than in normal people [19]. The exact mechanism behind this association is still unknown, but there have been some accepted theories. One such theory is based on Trimethylamine (TMA), produced when the gut microbiota metabolizes dietary choline, betaine, etc. The formation of Trimethylamine N-oxide (TMAO) after oxidation of TMA is the metabolite responsible for increasing the risk of CAD. This oxidation is facilitated by flavin mono-oxygenase (FMO)-3, which is a hepatic enzyme responsible for breaking down nitrogen-rich compounds derived from a non-vegetarian diet rich in phosphatidylcholine [20,21].

It has been found that high TMAO concentrations in blood positively correlate with increased chances of fat-laden plaque in blood vessels. TMAO is instrumental in activating Macrophage type-I and type-II class-A scavenger receptors (MSR-A). This results in the accumulation of low-density lipoproteins (LDL) due to increased receptivity and susceptibility to a plethora of microbiota that can easily bind to the receptor site. These steps act as ingredients for the formation of the plaque. TMAO has also been indicated to hinder bile acid formation, therefore further disrupting the cholesterol clearance pathway [20,22,23]. Therefore, it is hypothesized that gut microbiota is associated with CAD via the TMAO generation pathway. 

Hypertension

There are various risk factors like unhealthy lifestyle, diseases like diabetes, obesity, increased activity of the sympathetic system, etc., which may lead to the development of hypertension (HTN) [24]. With the help of its sympathetic nervous system, the body has its compensatory mechanisms by which blood pressure is maintained. These mechanisms include constriction of blood vessels, maintenance of normal physiological levels of electrolytes, and secretion of renin by the kidney [25]. The prolonged activation of the Renin-angiotensin-aldosterone system (RAAS) can lead to neuroinflammation in cardiovascular regulatory areas such as the paraventricular nucleus, rostral ventrolateral medulla, and nucleus of the solitary tract. This neuroinflammation is due to prorenin which facilitates hypothalamic microglial activation as seen in mice and spontaneously hypertensive rats (SHR). The above-mentioned cardiovascular regulatory areas are crucial for the regulation of sympathetic outflow, and their inflammation could further aggravate hypertension [26-28].

**Figure 1:**
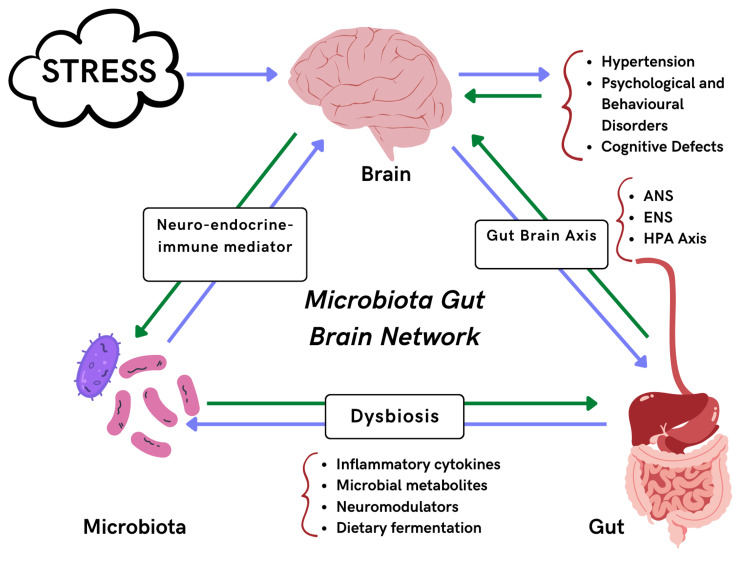
Relationship between gut microbiota and hypertension ANS: Autonomic nervous system; ENS: Enteric nervous system; HPA: Hypothalamic-pituitary-adrenal axis The figure is created by the author.

Inflammatory cytokines and microbial metabolites released by the gut microbiota could cause neuroinflammation in the cardiovascular regulatory areas. Therefore, a gut-brain axis is formed, capable of sympathetic activation and instrumental in the pathogenesis of hypertension, as explained above. The autonomic nervous system controls/regulates gut physiology like acid state, reception to pain, and maintenance of electrolyte levels [29]. The enteric nervous system (ENS), comprising the myenteric plexus and Meissner’s plexus, is responsible for ANS functions. This autonomous nervous system of the gut also called the ‘second brain’, passes signals to the brain with the help of the vagal nerve connected to the NTS. Studies have shown that gut microbiota such as *Roseburia*, *Bifidobacterium*, *Clostridium*, *Coprococcus*, *Oscillibacter*, *Enterococcus*, *Faecalibacterium*, *Blautia*, *Synergistetes,* and *Butyrivibrio* are found in low numbers in HTN patients than in average population. On the other hand, species like *Klebsiella*, *Parabacteroides*, *Salmonella*, *Actinomyces*, and *Streptococcus* are in large numbers in HTN patients [30-32]. These species indulge in disrupting ENS-CNS interactions by activating enterochromaffin cells and releasing neurotransmitters responsible for gut reflexes and normal physiological functions. This might also lead to a plethora of events translating to more gut permeability along with increased levels of metabolites in the circulation [33-36]. Therefore, these factors lead to hypertension in patients with dysbiosis and mild systemic inflammation. Figure 1 describes the connection between hypertension and gut microbes in brief. 

Heart failure

Heart failure (HF) is a heart condition comprising a collection of clinical symptoms like difficulty in breathing, edema in limbs, orthopnea along with raised jugular venous pressures (JVP), resulting due to change in the architecture of heart musculature leading to abnormalities in the normal functions of the heart [37]. Based on the available literature, it can be said that there exists a relationship between bacterial species in the gut and heart failure due to the former’s immuno-inflammatory properties. The species known to establish this association include *Candida*, *Campylobacter,* and *Shigella* [38]. Reduced cardiac output due to HF leads to low blood supply to tissues and congestion in intestinal capillaries, culminating in intestinal ischemia and edema in HF patients. The inflammatory reactions in the gut comprise intestinal edema and reduced blood supply, leading to increased gut permeability, thereby paving the way for endotoxins like lipopolysaccharides secreted by the microbiota to enter the blood initiating systemic inflammation and production of cytokines and interleukins [39-42]. These further result in inflammation and dysfunction in myocardial musculature [43,44].

Metabolites like TMAO mentioned in CAD have also shown their participation in the development of HF. TMAO acts on the cardiac musculature and is potent enough to cause the release of various cytokines and signaling pathways, which further lead to disruption in ATP homeostasis and hinder normal physiological functions of a cardiac muscle cell. The mechanism is well understood above in TMAO’s role in CAD leads to plaque formation as well as becoming a risk factor for HF. TMAO can lead to hypertrophy of ventricles and fibrosis of cardiac musculature. Increased levels of TMAO in the blood serve as a potential biomarker for developing a heart failure condition [45].

**Figure 2:**
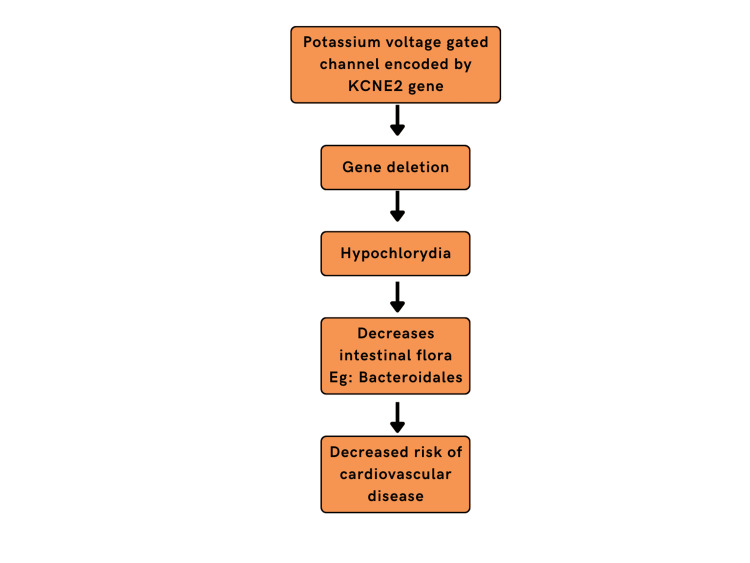
Effect of KCNE2 gene on heart failure The figure is created by the author.

Reduction in several certain species, specifically *Bacteroides* as well as *Bifidobacteria,* along with a rise in concentrations of *Firmicutes*, *Proteobacteria* contribute in steps associated with heart failure [46]. Recent molecular studies on animals have shown that deletion of certain cardiac-specific germlines, along with diet supplementation and therapies, might lead to lifespan extension. This novel scientific understanding is an outcome of research work done on a family of proteins that are potassium voltage-gated channels that are encoded by the KCNE2 gene. KCNE2 gene deletion leads to a state of Hypochlorhydria (low HCl levels) which has been shown to decrease certain intestinal flora like *Bacteroidales* known to contribute to the risk of cardiovascular disease. In peptic ulcers or gastroesophageal reflux disease (GERD) patients, when given proton-pump inhibitors (PPIs) like omeprazole to induce low acid levels, it was observed that the chances of developing HF are low as the responsible bacterial species concentration is lowered. The above correlation might help in designing novel therapies for the treatment of HF [47]. Figure 2 shows the effects of the KCNE2 gene on heart failure. 

Cyanotic congenital heart disease (CCHD)

According to a study, it is assumed that the gut of a fetus is free from microbiota and is not inhabited by any microbial species until the rupture of fetal membranes. The pioneer group of species from the mother’s genital and urinary tracts rushes to settle in a neonate just after full-term delivery. Various factors influence the initial gut microbiota, including breastfeeding, antibiotics, and other external factors. Usually, species from *Firmicutes*, *Bacteroidetes*, *Actinobacteria,* and *Proteobacteria* are observed [48].

Congenital heart disease (CHD) occurs during the embryological period leading to structural malformations in the cardiac system as well as the vascular system. It is the top contributor to neonatal mortalities. Cyanotic CHD is one type which comprises mainly right-sided obstructive lesions (Tetralogy of Fallot, Critical pulmonic stenosis, Ebstein’s anomaly), left heart obstructive lesions (Critical aortic stenosis, Coarctation of the aorta, Interrupted aortic arch) and some lesions like transposition of the great arteries, Tricuspid atresia and Truncus arteriosus [48-51].

According to the PubMed database, no study was found that explicitly outlines the intestinal microbiota present in a neonate's gut with CCHD [48].

Necrotizing enterocolitis (NEC) incidence is the highest among premature infants, making it the topmost medical emergency in the gastrointestinal department [52]. NEC allows opportunistic microbiota to express themselves by its antigens from the gut lumen, thereby initiating an inflammatory response resulting in gut necrosis and increasing chances of cardiac instability [53,54].

**Figure 3:**
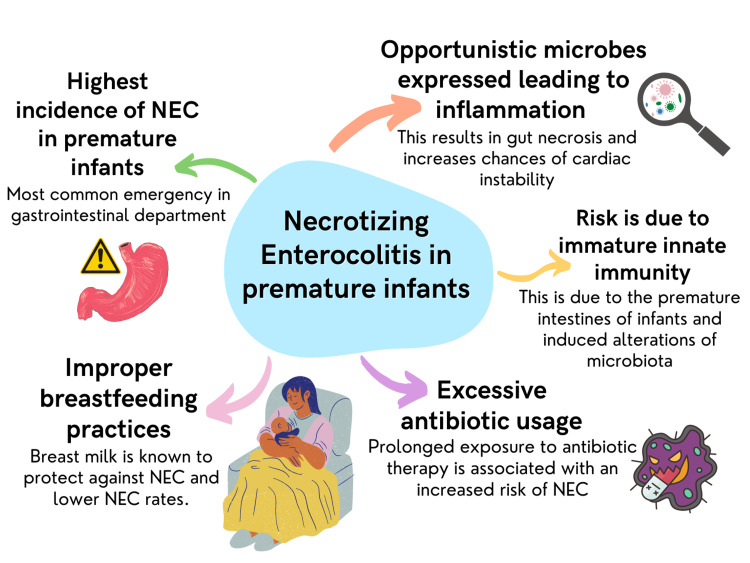
Necrotizing enterocolitis in premature infants NEC: Necrotizing enterocolitis The figure is created by the author.

NEC risk in a premature infant is mainly due to immature innate immunity of premature intestines and induced alteration of microbiota, probably resulting from excessive antibiotic usage, improper breastfeeding practices, and being prone to opportunistic hospital infections [55]. Figure 3 displays the spectrum of problems associated with Necrotizing enterocolitis in premature infants. 

Atrial fibrillation

Atrial fibrillation (AF) is characterized by ineffective and uncoordinated atrial contraction. AF is a life-threatening condition worldwide, with a lifetime risk of 26% and 23% for men and women who have crossed 40 years [56]. The blood flow from the ventricles decreases, bringing in a series of symptoms like heart failure (HF), breathlessness, syncope, and cardiac arrest [56]. The human gut serves as a reservoir for microbiota, which can influence the heart’s function through various metabolites or the immune system [57]. *Firmicutes*, *Bacteroidota*, *Fusobacteriota*, *Proteobacteria,* and *Actinobacteria,* are known to be responsible for playing a role in the development of AF. A cohort study observed that patients with atrial fibrillation and control groups shared 1,189 operational taxonomic units (OTUs). Within them, 897 specific OTUs were observed exclusively in patients having atrial fibrillation, whereas only 477 were observed only in controls [58].

*Haemophilus*, *Alistipes*, *Enterococcus*, *Weissella*, *Parabacteroides*, *Megamonas*, *Streptococcus*, and *Klebsiella* were in high concentration in patients with atrial fibrillation. *Agathobacter* concentrations were observed to be higher in the control group [58].

Gram-negative bacteria have endotoxins like lipopolysaccharides on their outer membrane. Lipopolysaccharides, as understood by their name, are endotoxins formed of lipids and polysaccharides. Therefore, it can be assumed that if the population of bacteria increases, endotoxins associated with them will also increase. A positive correlation is observed between the rise of gram-negative bacteria and the production of lipo-polysaccharides (LPS). Rising concentrations of endotoxins lead to inflammation by the activation of NLRP3 (inflammasomes). Inflammasomes are part of innate immunity and act as a line of defense when any pathogenic microbe is encountered [59]. These inflammasomes act by caspase-1 activation, leading to the discharge of interleukins like IL-1β and IL-18. Interleukins change the permeability of the intestine, facilitating endotoxin release from the gut to circulation. This cycle continues resulting in the release of cytokines, endotoxins, etc., damaging the gut permeability and accumulation of LPS in the bloodstream. Consequently, with rising LPS, Tol-like receptors (especially, TLR-4) acting as sensors for any rise in pathogens results in activation of nuclear factor-kappaB (NF-κB). NF-κB codes for the release of a large number of cytokines, and its activation would result in their expression. These long chains of events result in lipid collection in vascular structures. Lipid accumulation ensues upregulation of interleukins like IL-6, IL-8, chemo-tactic proteins of monocytes, and cell adhesion molecules (CAMs), causing inflammation in vascular structures. Inflammation is characterized by myocyte apoptosis, fibrosis, and enlargement [60,61]. Therefore, it can be said that there is significant evidence that proves the association of gut microbiota with atrial fibrillation.

Rheumatoid heart disease

Rheumatoid heart disease (RHD) is a condition arising when the heart’s valves are damaged, leading to cardiovascular complications [62]. The most affected valves in RHD patients are the Mitral (bicuspid) valves [63]. RHD is characterized by an autoimmune response elicitated by the body when there is a *Streptococcal* (group A *Streptococcus* (GAS)) infection [62]. A cohort study was performed to identify gut-related microbial changes in RHD patients. The microbiota in the fecal matter was extracted for examination from healthy people and RHD patients. It was revealed that certain gut species like *Eubacterium* along with *Bacteroides* residing in the gut negatively correlate with Left atrial diameter (LAD) [64]. *Neisseria*, *Pelomonas*, *Ralstonia*, *Acinetobacter*, *Streptococcus*, *Thermus*, *Sphingomonas*, *Agrobacterium*, *Rothia*, *Prevotella*, *Shigella*, *Fusobacterium*, *Afipia*, *Burkholderia,* and *Caulobacter* are the 15 most common genera known to affect bicuspid heart valves of RHD patients [64]. The relative concentration of *Faecalibacterium* and *Bacteroides* was fundamentally diminished. Instead, it was found that *Gemmiger*, *Ruminococcus*, *Shigella*, *Bifidobacterium*, *Dorea,* and Streptococcus species proliferated in healthy as well as RHD patients. Gut microbial taxonomy serves as a good marker for discrimination of patients suffering from RHD and the healthy control group [64].

The foundation of treatment in RHD is a penicillin-based treatment for intense rheumatic fever. If the condition doesn’t improve, surgical intervention is the last option for the repair/replacement of impaired heart valves. The chances of a positive outcome by surgical intervention increase if prior immunization was administered [62,65,66].

Stroke

Ischemic stroke is a condition when there is an obstruction in cerebral blood flow, mainly due to thrombus formation or embolization. Stroke is of two types ischemic stroke and hemorrhagic stroke. Around 87% of the total incidence of stroke is acute ischemic stroke [67]. The CNS regulates certain aspects of the physiology of the digestive system. It controls the motility of the gut, secretions by different cells, and mucosal immune response. In the gut, the microbial populace has been observed to correlate with the central nervous system (CNS) through neurological and immunological pathways. Therefore, a two-way directional neural-gut axis is formed [68]. The intestinal nervous system and the neuronal-glial-epithelial system coordinate the neural gut axis [69-71]. Catecholamines and hormones act on gut microbiota which in turn produce gut microbiota neuromodulators and neurotransmitters [72-74].

A study on a mouse model revealed that certain microbial species traveled from the gut to the brain and occluded the middle cerebral artery (MCA), causing stroke [75]. After the stroke episode, the blood-brain barrier (BBB) allows entry of the immunity cells (regulatory T-cells, IL-17, Th17 cytokines, etc.) to suppress bacterial infection in the MCA. The interaction between the pathogenic gut microbiota and immune cells leads to inflammation. This inflammation contributes to occlusion and further deteriorates the stroke condition [76-78].

In another study, a cerebral tumor acquired the ability to alter normal digestive physiology as well as gut immunity by influencing the autonomic nervous system (ANS) and the hypothalamic-pituitary-adrenal organs (HPA). It has been observed that within 24 hours of a sudden stroke episode, B and T cells present in Peyer’s patches are diminished [79]. Due to declining levels of B and T cells, the body incorporates a compensatory mechanism that involves activation of GALT (gut-associated lymphatic system) along with increased immune responses by the gut microbiota. In this case, gut microbiota proved to be beneficial and contributed to the host’s defense mechanism [79]. 

Vascular changes

A study on patients suffering from high blood pressure showed high amounts of intestinal Fatty-acid binding protein (I-FABP), augmented helper T17 cells, and (LPS) lipo-polysaccharide. They cause inflammation in the intestine and increase permeability [80]. The increased permeability allows gram-negative bacteria to enter the blood circulation. Gut microbes, commonly *Bacteroidetes* and the *Firmicutes,* are observed to be beneficial in the morphogenesis of epithelial cells of the gut [81-83]. Gut microbes produce short-chain fatty acids (SCFAs) and uremic toxins, which can severely damage the functioning of the endothelium. Uremic toxins are hippuric acid, indoxyl sulfate, and phenyl sulfate. This help generates ROSs (reactive oxygen species), thus promoting endothelial dysfunction [84]. The inducible nitric oxide synthase (iNOS) produces a high quantity of nitrous oxide. Nitrous oxide is activated by the LPS (lipopolysaccharide), which, when reacted with free radicals of oxygen, makes peroxynitrite, causing constriction of vessels (vasoconstriction effect) [85].

Paneth cells present in the crypts of Lieberkühn of the small intestine are responsible for maintaining the correct concentration of the commensal microbes. Since the initial colonization of the gut by the microbes, Paneth cells' role is irreplaceable as it is responsible for the regulation of the microbiota populace by the secretion of antimicrobial proteins and peptides. Therefore, it can be said that Paneth cells are regulators of the microvasculature of the intestine [86].

There are various central signaling pathways like the Hedgehog pathway [87], the Wingless-related integration site (WNT) pathway, the Notch pathway, the transforming growth factor-β/Smad pathway, and tyrosine kinase pathways involved with intestinal morphogenesis during the embryological period. The ecological environment of the gut in the intestinal mucosa is a part of the above-mentioned multiple pathways for gut morphogenesis [88]. A study showed that protease-activated receptor-1 (PAR-1) influences and regulates the pathways and is, therefore, instrumental in the architecture of minute capillaries in villi of the small intestine [89].

Microbiota-based CVD therapy

Since establishing a relationship between gut microbiota and CVDs, new therapies have evolved to treat cardiovascular diseases. The most effective therapy changes in diet and lifestyle that appease the beneficial microbes. The use of probiotics, judicious use of antibiotics, and fecal microbiota transplantation are novel approaches in the treatment of CVD’s keeping the gut microbiota factor in view. However, still, further experiments are needed to concrete the concept of microbiota-based CVD therapy [90].

Research gap

Although there exists a plethora of research studies that establish the relationship between gut microbiota and increased risk of cardiovascular diseases, only a handful of studies contribute to providing evidence that indicates the direct participation of gut microbiota in the pathogenesis of CVDs. Large sample size studies are needed to understand the deep connection of this relationship between the two components. Knowledge is limited in understanding specific interactions of two microbial species, their interaction with the human body, and how these species play a role in disease manifestation. These topics contribute to significant research gaps. 

## Conclusions

The human gut consists of vast strata of microbiota which almost act like a silent organ. Some of them are beneficial, some are neutral, and some have pathogenic potential. Based on the available literature, there is significant evidence proving an association of gut microbiota with cardiovascular diseases. Dysbiosis is observed in patients with coronary artery disease, hypertension, heart failure, and other heart conditions. According to studies, various metabolites secreted by microbiota lead to the worsening of cardiac conditions. Therefore, it is high time that treatment modalities based on gut microbiota should be considered. This can be achieved very easily by modification in diet, lifestyle, and correct usage of probiotics and supplements.

Further depth in the concept of association between gut microbiota and CVDs can be only made possible by more experiments and research studies, as this is only the tip of the iceberg. The focus should be on outlining direct pathways and metabolites that influence the development of CVDs. While developing newer drugs, their action on gut microbiota should also be considered. Preserving and maintaining beneficial microbiota has a positive impact not only on cardiac diseases but also on the general physiology of the human body. Therefore, scientists should focus on ideas and concepts that might help improve the status of microbiota residing in the intestines, which seems to have the potential of providing a new approach to treating cardiovascular diseases.
